# Protection from Endotoxic Uveitis by Intravitreal Resolvin D1: Involvement of Lymphocytes, miRNAs, Ubiquitin-Proteasome, and M1/M2 Macrophages

**DOI:** 10.1155/2015/149381

**Published:** 2015-01-15

**Authors:** S. Rossi, C. Di Filippo, C. Gesualdo, N. Potenza, A. Russo, M. C. Trotta, M. V. Zippo, R. Maisto, F. Ferraraccio, F. Simonelli, M. D'Amico

**Affiliations:** ^1^Multisciplinary Department of Medical-Surgical and Dental Specialities, Second University of Naples, Via Pansini 5, 80131 Naples, Italy; ^2^Section of Pharmacology “L. Donatelli”, Department of Experimental Medicine, Second University of Naples, Via Costantinopoli 16, 80138 Naples, Italy; ^3^DiSTABiF, Second University of Naples, Via Vivaldi 43, 81100 Caserta, Italy; ^4^Department of Clinical, Public and Preventive Medicine, Second University of Naples, Via Armanni 5, 80138 Naples, Italy

## Abstract

This study investigated the protective effects of intravitreal Resolvin D1 (RvD1) against LPS-induced rat endotoxic uveitis (EIU). RvD1 was administered into the right eye at a single injection of 5 *μ*L volume containing 10–100–1000 ng/kg RvD1 1 h post-LPS injection (200 *μ*g, *Salmonella minnesota*) into thefootpad of Sprague-Dawley rats. 24 h later, the eye was enucleated and examined for clinical, biochemical, and immunohistochemical evaluations. RvD1 significantly and dose-dependently decreased the clinical score attributed to EIU, starting from the dose of 10 ng/kg and further decreased by 100 and 1000 ng/kg. These effects were accompanied by changes in four important determinants of the immune-inflammatory response within the eye: (i) the B and T lymphocytes, (ii) the miRNAs pattern, (iii) the ubiquitin-proteasome system (UPS), and (iv) the M1/M2 macrophage phenotype. LPS+RvD1 treated rats showed reduced presence of B and T lymphocytes and upregulation of miR-200c-3p, miR 203a-3p, miR 29b-3p, and miR 21-5p into the eye compared to the LPS alone. This was paralleled by decreases of the ubiquitin, 20S and 26S proteasome subunits, reduced presence of macrophage M1, and increased presence of macrophage M2 in the ocular tissues. Accordingly, the levels of the cytokine TNF-*α*, the chemokines MIP1-*α* and NF-*κ*B were reduced.

## 1. Introduction

Uveitis is an inflammation of the uveal tract including the iris, ciliary body, and choroid. This disease can be idiopathic or associated with infectious and systemic disorders and can be classified anatomically into either anterior, intermediate, and posterior or panuveitis and as acute or chronic disease, depending on whether it lasts more or less than 3 months in duration [1]. The inflammation may cause a permanent damage in various ocular tissues with visual impairment for macular edema, optic nerve dysfunction, vitreous opacification, and cataract formation [2]. Although, the exact pathogenesis of uveitis is not clearly described, it is well known that the mediators of immune-inflammatory responses are responsible for it [3]. Recently, Rossi et al. [4] demonstrated that the systemic injection of the lipid-derived protein Resolvin D1 (RvD1), potent mediator that promotes the resolution of the inflammatory response back to a noninflamed state [3, 5–8], is able to counteract the insurgence of uveitis by improving the immune-inflammatory profile of the external and median tunics of the eye despite the presence of the blood-ocular barrier which may have limited the concentration of the RvD1 achieved within the vitreous and chorioretina. The purpose of the present study was to further elucidate the mechanisms of RvD1 protection by injecting the protein directly into the vitreous, and four important determinants of the immune-inflammatory response within the eye were monitored: (i) the B and T lymphocytes; (ii) the ocular miRNAs pattern; (iii) the ubiquitin-proteasome system (UPS); and (iv) the macrophage phenotype.

## 2. Material and Methods

### 2.1. Induction of EIU

Male Sprague-Dawley rats (180–220 g) were injected in one footpad with 200 *μ*g of lipopolysaccharide (LPS,* Salmonella minnesota*, Sigma, St Louis, MO, USA) in 0.1 mL of sterile pyrogen-free saline for the induction of EIU. 1 h following LPS treatment RvD1 (Cayman Chemical, MI, USA) was intravitreally injected into the right eye at the dose of 10-100-1000 ng/kg, chosen in the range of those used in murine models of inflammation [4, 9]. Intravitreal injection was made as described previously with some modifications [10, 11], rats were anesthetized by intraperitoneal injection of pentobarbital (45 mg/kg in saline), and pupils were dilated by instillation of one drop of tropicamide 5% and had one drop of tetracaine 1% administered for local anaesthesia. RvD1 was injected once using sterile syringes fitted with a 30-gauge needle containing 5 *μ*L [10, 12] of reconstituted RvD1 solution. The following experimental 5 groups were considered (*n* = 6 rats for each group): vehicle (saline+ethanol); saline+LPS; and LPS+RvD1 at the doses of 10-100-1000 ng/kg. Rats were killed 24 h after each treatment.

### 2.2. Clinical Score Attributed to EIU

Animals were examined with a biomicroscope 24 h after vehicle, LPS, or LPS+RvD1 (10-100-1000 ng/kg) treatment. Clinical manifestations of EIU were graded from 0 to 4 in a blinded fashion according to the previously reported scoring system [4]: 0 = no inflammatory reaction; 1 = discrete dilation of iris and conjunctival vessels; 2 = moderate dilation of iris and conjunctival vessels with moderate flare in the anterior chamber; 3 = intense iridal hyperemia with intense flare in the anterior chamber; and 4 = same clinical signs as 3 with presence of fibrinoid exudation in the pupillary area and miosis. No signs of uveitis were observed in the animals at the beginning of each experiment. Clinical EIU was considered positive when the score assigned was >1. EIU clinical data shown were representative of 6 experimental groups and presented as mean ± SEM of 6 observations for each group.

### 2.3. Eye Samples

After 24 h of EIU, the eyes were harvested and cut in two halves. One half of each eye was immediately frozen in liquid nitrogen and stored at −80°C for the later biochemical assays described below. The other half of each eye was immediately fixed by immersion in 10% buffered formalin and paraffin-embedded for immunohistochemistry. Sections were serially cut at 5 *μ*m, placed on lysine-coated slides, and stained with hematoxylin and eosin and with the trichrome method.

### 2.4. Purification of Total RNA from Ocular Tissue

After thawing, the samples were placed in dry ice and then an appropriate volume of PBS (phosphate-buffer saline) was added, in order to remove any residues and impurities that could interfere with the determination of their weight. Then, the correct volume of lysis buffer (QIAzol Lysis Reagent), required for the tissue homogenization, was determined. The homogenization was performed using the Potter homogenizer. Total RNA, including small RNAs, was extracted using the MiRNeasy Minikit (Qiagen), according to the manufacturer's protocol. Before the extraction, Syn-cel-miR-39 miScripit miRNA Mimic 5 nM was added to each sample, in order to monitor the efficiency of miRNA isolation. Total RNA was extracted from 200 *μ*L of tissue lysate and then eluated in Rnase free water. The quality and quantity of the RNA were evaluated by 260/280 ratio using NanoDrop spectrophotometry.

### 2.5. Reverse Transcription of Total RNA

Mature miRNAs were converted in cDNA with a reverse transcription reaction carried out using the MiScript II Reverse Transcription Kit (Qiagen) according to the manufacturer's protocol.

### 2.6. Real-Time PCR for Mature miRNA Expression

cDNA prepared in a reverse transcription reaction using miScript HiSpec Buffer served as the template for real-time PCR analysis using the Rat Inflammatory Response & Autoimmunity miRNA PCR Array (MIRN-105Z) (which contained miRNA-specific miScript Primer Assays); the miScript SYBR Green Kit, which contained the miScript Universal Primer (reverse primer) and QuantiTect SYBR Green PCR Master Mix. The qRT-PCR analysis was performed on a MyiQ2 thermocycler (Bio-Rad).

### 2.7. Immunohistochemistry

Paraffin-embedded eye samples were treated with a xylene substitute (Hemo-De; Fisher Scientific) in order to remove the paraffin, and tissue sections were rehydrated with ethanol gradient washes. Tissue sections were quenched sequentially in 3% hydrogen peroxide aqueous solution and blocked with PBS 6% nonfat dry milk (Biorad, Milan, Italy) for 1 h at room temperature. Sections were then incubated with specific antibodies anti CD20^+^ B cell, anti-CD4^+^ T lymphocytes, and anti-ubiquitin (Santa Cruz Biotec, USA). M1 macrophage phenotypes were characterized by the expression of anti-integrin alpha X/CD11c antibody (Abcam, Cambridge, UK) and for the macrophages M2 phenotype expression an anti-mannose receptor antibody CD206 (Abcam, Cambridge, UK). Sections were washed with PBS and incubated with secondary antibodies. Specific labelling was detected with a biotin-conjugated goat anti-rabbit IgG and avidin-biotin peroxidase complex (DBA, Milan, Italy). For each immunohistochemical experiment, a negative control was performed with the primary antibody omitted (data not shown). The specimens were analyzed by an expert pathologist (intraobserver variability 6%) blinded to the experimental protocol. Six distinct tissue sections for each group of animals were done and 23 microscopic fields were analyzed in each section for a total area of of 4.3623*e* + 005 *μ*m^2^ at 400x magnification. Of each total area a computer-aided planimetry (IM500, Leica Microsystem, Milano, Italy) was performed and the percentage of positive stained area per total area analyzed calculated. A color threshold mask for immunostaining was defined and applied to all sections.

### 2.8. Western Blotting Assay

Frozen tissues were homogenized in a solution containing 0.5% hexadecyl-trimethyl-ammonium bromide dissolved in 10 mM potassium phosphate buffer (pH 7) and centrifuged for 30 min at 4,000 ×g at 4°C. Tissues protein concentration was measured by the Bradford method (1976); then, 15 *μ*g protein sample was used for the gel electrophoresis in a 6% PAGE separation gel. The samples were electrotransferred onto a PVDF membrane. Blots were blocked with 5% nonfat dry milk for 1 h at room temperature and then incubated with primary specific antibodies overnight, followed by incubation with a horseradish peroxidase-conjugated secondary antibody for 1 h at room temperature. The signal was normalized to the intensity of a housekeeping protein and expressed as densitometric unit (DU). Western Blots were performed to evaluate the expression of the UPS system (20S and 26S proteasome subunits), NF-*κ*B (p50, p65, and p105 subunits). The following primary antibodies purchased by Santa Cruz (USA) were used: anti-proteasome subunit (Fl-76, anti 20S, and anti 26S), NF-*κ*B p65 (C-20), NF-*κ*B p50, and p105 (H-119). For all assays secondary antibodies HRP horseradish peroxidase were used: donkey polyclonal-rabbit IgG, goat anti-mouse, goat anti-rabbit, and were all purchased by Santa Cruz (USA).

### 2.9. ELISA Assay

Tumor necrosis factor alpha (TNF-*α*) and macrophage inflammatory protein 1 alpha (MIP1-*α*) levels were determined in ocular tissues using a commercially available ELISA purchased from R&D Systems (Abingdon, UK). For example, tissue supernatant aliquots (50 *μ*L) were assayed for MIP1-*α* and compared to a standard curve constructed with 4.7–150 pg/mL of chemokine. The ELISA showed negligible (<1%) cross-reactivity with several murine cytokines and chemokines (data as furnished by manufacturer).

## 3. Statistical Analysis

Data analysis was performed with the web-based software package (http://pcrdataanalysis.sabiosciences.com/mirna/arrayanalysis.php) for the miRNA PCR array system. The amplification curves were analyzed using the ΔΔCT-method of relative quantification, in order to obtain specifics miRNA expression patterns in each treatment, and then to compare the different profiles. Snord68, a small nucleolar RNA, was used for normalization of qRT-PCR results. DCt value for each miRNA profiled in a plate is calculated using the formula DCt = Ct^miRNA^ − Ct^cel-SNORD68^. DDCt for each miRNA across 2 groups of samples is calculated using the formula: DDCt = DCt of treatment group − DCt of control group. Expression fold change was then obtained as 2^−DDCt^ (the normalized gene expression (2^−DCt^) in the treatment group divided the normalized gene expression (2^−DCt^) in the control group). Data are reported as fold regulation, where fold regulation is equal to the fold change for fold change values >1 (upregulation), while for fold change values <1 (downregulation) it is the negative inverse of the fold change. The *P* values are calculated based on Student's *t*-test of the replicate 2^−DCt^ values for each miRNA in the control and treatment groups. The criteria of differential expression were *P* < 0.05 and *P* < 0.01.

Other values are expressed as mean ± SEM of *n* number of rats for the* in vivo* experiments. Statistical analysis was assessed either by Student's *t*-test (when only two groups were compared) or one-way ANOVA followed by Dunnett's test (more than two experimental groups). A probability *P* value less than 0.05 was considered significant to reject the null hypothesis.

## 4. Results

### 4.1. Intravitreal RvD1 Improves Clinical Score of EIU

**Figure 1:**
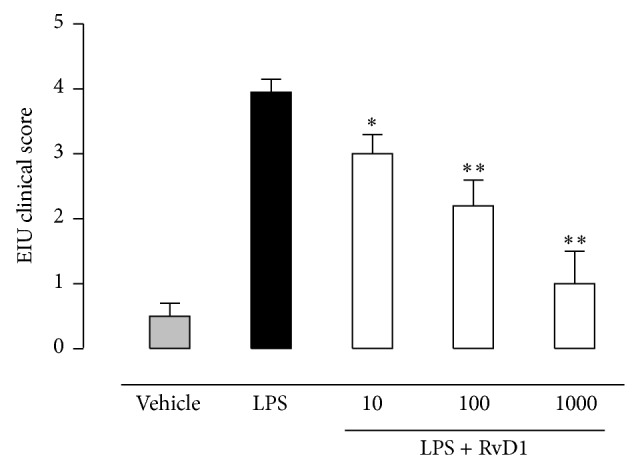
Intravitreal Resolvin D1 (RvD1) improves the clinical score in rats with EIU. The rats were treated with vehicle (saline+ethanol), LPS (200 *μ*g/rat), and LPS+RvD1 at the dose of 10-100-1000 ng/kg 1 h post-LPS treatment and were evaluated 24 h after injections. Clinical manifestations of EIU were graded as reported in test (see Section 2). Values are reported as the mean ± SEM, of *n* = 6 observation for each experimental group. ^*^ *P* < 0.05 and ^**^ *P* < 0.01 compared with LPS-treated group.

**Figure 2:**
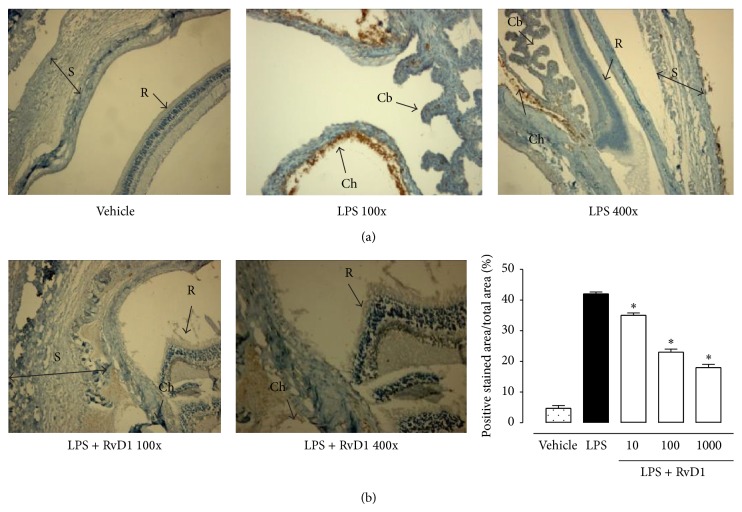
Intravitreal Resolvin D1 (RvD1) reduces CD4^+^ immunostaining. (a) Representative immunohistochemistry of ocular tissues showing that treatment with RvD1 decreases immunostaining for CD4^+^ T cells, already significant at the lowest dose (10 ng/kg, 1 h post-LPS treatment) with respect to the LPS treated rats. (b) Graph showing the percentage of the total positive stained area for CD4^+^ per total area analyzed at 400x magnification. Values are mean ± SEM of *n* = 6 observation for each group. ^*^ *P* < 0.05 and ^**^ *P* < 0.01 versus LPS-treated group. R = retina; S = sclera; Ch = Choroid; Cb = ciliary bodies.

**Figure 3:**
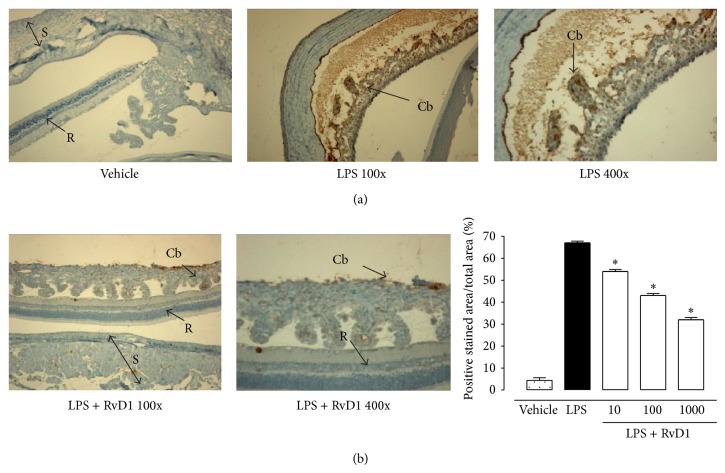
Intravitreal Resolvin D1 (RvD1) reduced CD20^+^ immunostaining. (a) Representative immunohistochemistry showing that intravitreal RvD1 decreased immunostaining for CD20^+^ B cell, already significant at the lowest dose (10 ng/kg, 1 h post-LPS treatment) with respect to the LPS treated rats. (b) Graph showing the percentage of the total positive stained area for CD20^+^ per total area analyzed at 400x magnification. Values are mean ± SEM of *n* = 6 observation for each group. ^*^ *P* < 0.05 and ^**^ *P* < 0.01 versus LPS-treated group. R = retina; S = sclera.

24 h after the administration of 200 *μ*g LPS into the footpad of Sprague-Dawley rats, severe changes of the structure of the eye occurred, with a clinical score of 3.95 ± 0.2 attributed (Figure 1). Ocular tissues of LPS treated rats were largely edematous and telangiectasic with an oblong profile of the blood vessels and markedly positive for CD20^+^ B cells, CD4^+^ T lymphocytes (Figures 2 and 3).

In contrast, intravitreal RvD1 injection (at doses of 10-100-1000 ng/kg) improved clinical score attributed to EIU (Figure 1) and showed a strong attenuation of the immune processes as highlighted by the decrease of the percentage of the area stained for CD4^+^ T lymphocytes, CD20^+^ B cells into the sclera, choroid, retina, and ciliary bodies (*P* < 0.01) (Figures 2 and 3). It is to note that intravitreal injection of RvD1 in LPS treated rats decreases significantly the immune-inflammatory reaction already at the doses of 10 ng/kg (*P* < 0.05 versus LPS treated rats) (Figures 2 and 3).

### 4.2. RVD1 and miRNA Profile

**Figure 4:**
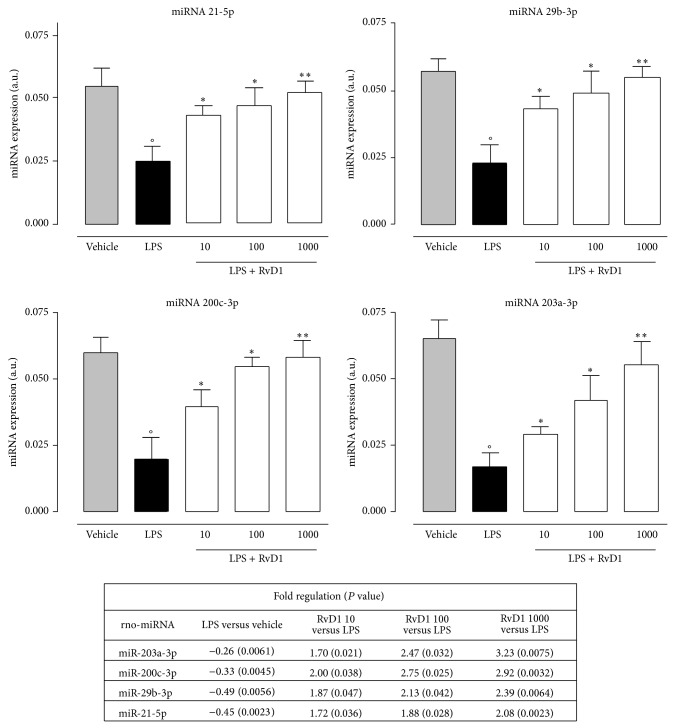
Change in miRNAs expression in LPS and in LPS+RVD1 treated rats. 4 miRNAs were significantly upregulated in LPS+RvD1 (10-100-1000 ng/kg) compared to LPS-rats. Arbitrary units are 2^−Dct^ values obtained from RT-qPCR analysis and without any multiplying factor. Values are mean ± SEM of *n* = 6 observations for each groups. °*P* < 0.01 versus vehicle-group; ^*^ *P* < 0.05 and ^**^ *P* < 0.01 versus LPS-treated group. The table shows the miRNAs fold regulation and relative *P* values.

**Figure 5:**
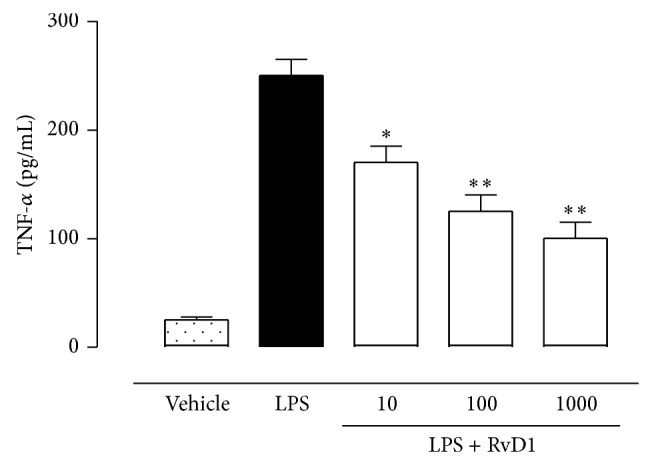
Resolvin D1 (RvD1) and TNF-alpha. ELISA for TNF-alpha (TNF-*α*) in ocular tissues of vehicle, LPS, and LPS+RvD1 (10-100-1000 ng/kg) treated rats as reported in materials and methods. Values are mean ± SEM of *n* = 6 observation for each group. ^*^ *P* < 0.05 and ^**^ *P* < 0.01 versus LPS treated rats.

**Figure 6:**
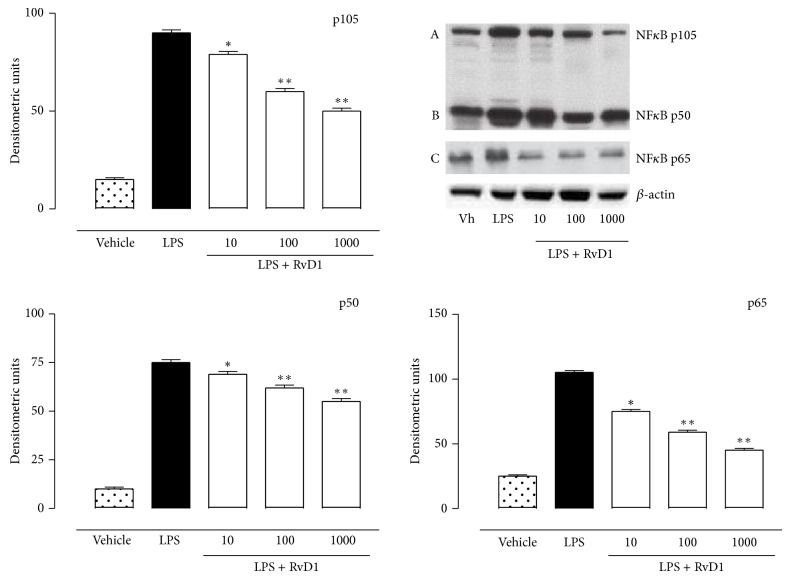
Western blotting analysis for NF-*κ*B. Western blotting technique showed that injection of resolvin D1 (RvD1, 10-100-1000 ng/kg) into the vitreous of LPS-treated rats reduced the expression of activated NF-*κ*B: p50, p65, and p105. Results are expressed as densitometric units and represented the mean ± SEM of *n* = 6 observation for each group. Vh = vehicle; ^*^ *P* < 0.05 and ^**^ *P* < 0.01 versus LPS-treated rats.

The characterization of miRNAs profile of LPS+RvD1-treated rats was obtained by comparing these rats with vehicle+LPS-treated rats. The analysis based on their fold changes showed a significant (*P* < 0.05) upregulation of miR-200c-3p (predicted to regulate IL-13 and VEGF-alpha), miR203a-3p (predicted to regulate IL-24 and PRKC*α*), miR29-3p (predicted to regulate TNFRS1A), and miR-21-5p (predicted to regulate NFk-B activity), in ocular tissues of LPS+RvD1-treated rats compared to the vehicle+LPS group (Figure 4). Interestingly, upregulation of miR29-3p and miR-21-5p induced by RvD1, significant already at the lowest dose of 10 ng/kg, was concomitant with the decrease of TNF-*α* and NF-*κ*B levels in the ocular tissue (Figures 5 and 6). Particularly, the expression of NF-*κ*B, as reflected by the selective analysis of the activated forms p50, p65, and p105, was significantly lower in ocular tissues of LPS+RvD1 (10-100-1000 ng/kg) treated rats with respect to LPS alone (Figure 6).

### 4.3. Intravitreal RvD1 Treatment and the Ubiquitin-Proteasome System

**Figure 7:**
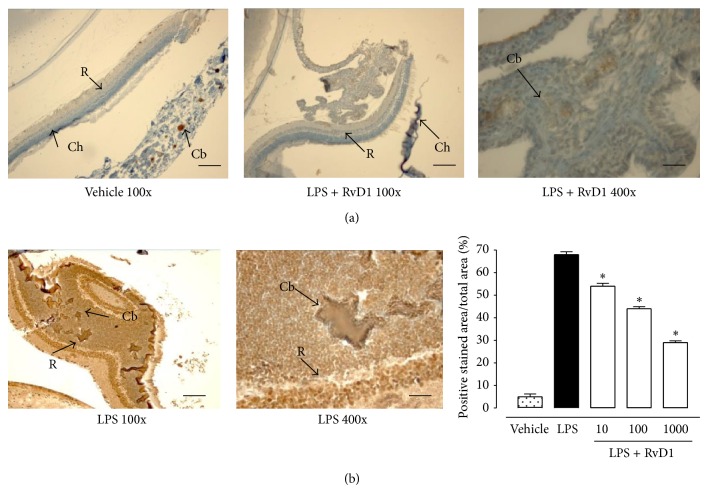
Resolvin (RvD1) and ubiquitin. (a) Sections showing representative immunohistochemistry for ubiquitin in the ocular tissues of rats treated with vehicle (saline+ethanol), LPS (200*μ*g/rat), or resolvin D1 at the lowest dose (10 ng/kg, 1 h post-LPS). (b) Graph showing the percentage of positive stained area for ubiquitin per total area analyzed at 400x magnification. Values are mean ± SEM of *n* = 6 observations for group. ^*^ *P* < 0.01 versus LPS-treated rats. R = retina; S = sclera; Cb = ciliary bodies; Ch = choroid.

**Figure 8:**
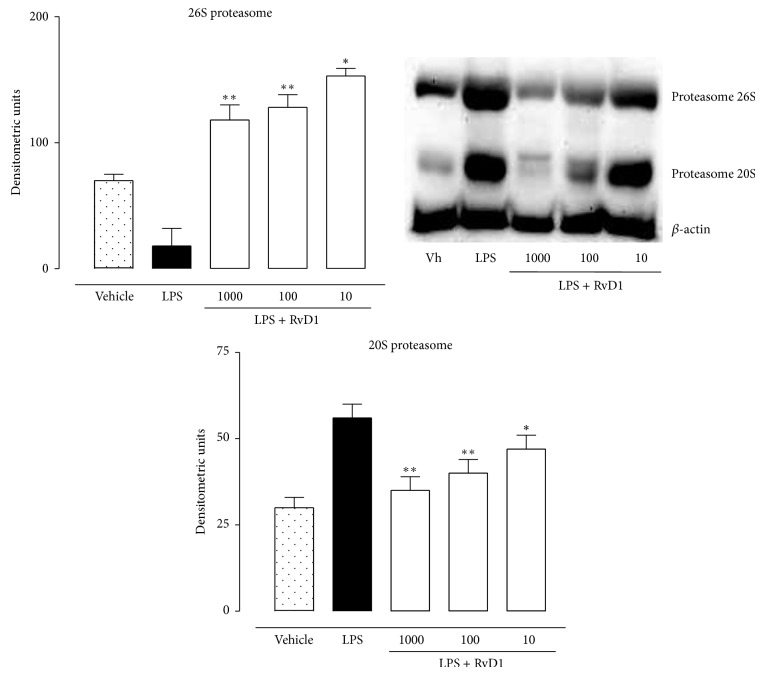
Western Blotting showing that Resolvin D1 (RvD1) treatment reduces the ocular proteasome system. Ocular tissue homogenates from the eyes of LPS-treated rats showed highest levels of 20S and 26S proteasome subunits. Intravitreal RvD1 (10-100-1000 ng/kg) post-LPS decreased the levels of the proteasome subunits with the respect to LPS alone. Results are expressed as densitometric units and represented the mean ± SEM of *n* = 6 observation for each group. Vh = vehicle; ^*^ *P* < 0.05 and ^**^ *P* < 0.01 versus LPS.

Ocular tissue of LPS treated rats showed an increase of the ubiquitin-proteasome levels that was reduced by RvD1 (see Figure 7 for ubiquitin immunohistochemistry and Figure 8 for proteasome western blotting). Indeed, 20S and 26S proteasome subunits were found significantly reduced (*P* < 0.05) already at a dose of 10 ng/kg (see Figures 7 and 8).

### 4.4. Intravitreal RvD1 Treatment Induced Change in Macrophage Phenotypes and Decreased Chemokine MIP1-*α* Levels

**Figure 9:**
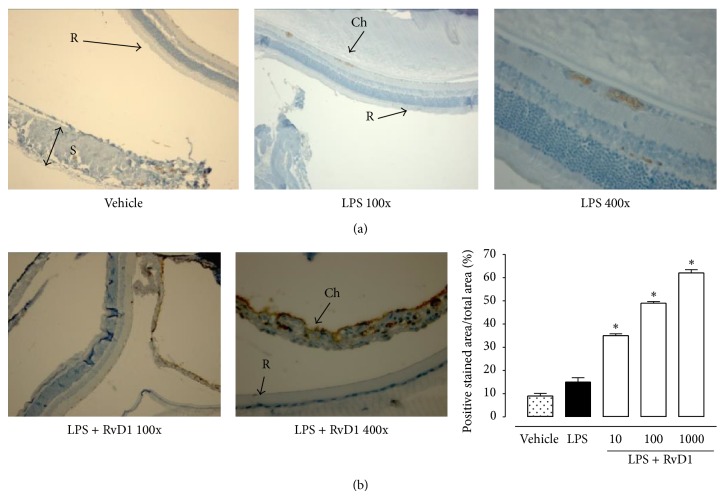
Resolvin D1 (RvD1) treatment induces M2 expression macrophage phenotypes. (a) Representative immunohistochemistry of eye tissues showing that treatment with resolvin D1 (RvD1, 10 ng/kg 1 h post-LPS) increased immunostaining for M2 macrophage phenotype expression an anti-mannose receptor antibody CD206. (b) Graph showing the percentage of positive stained area per total area analyzed at 400x magnification. Values are mean ± SEM of *n* = 6 observations for group. ^*^ *P* < 0.05 and ^**^ *P* < 0.01 versus LPS-treated group. R = retina; S = sclera Cb = ciliary bodies; Ch = choroid.

**Figure 10:**
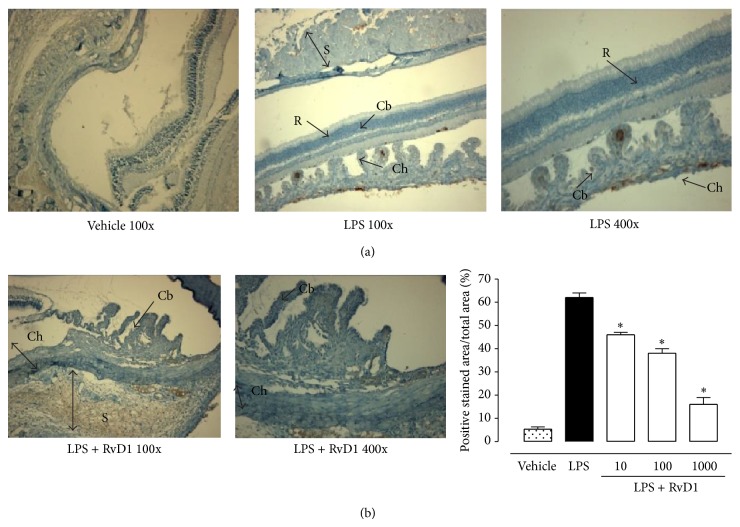
(a) Representative immunohistochemistry of eye tissues showing that treatment with resolvin D1 (RvD1, 10 ng/kg 1 h post-LPS) reduces immunostaining for anti-integrin alpha X/CD11c antibodies selective for M1 macrophages phenotype. (b) Graphs showing the percentage of positive stained area per total area analyzed at 400x magnification. Values are mean ± SEM of *n* = 6 observations for group. ^*^ *P* < 0.05 and ^**^ *P* < 0.01 versus LPS-treated group. R = retina; S = sclera Cb = ciliary bodies; Ch = choroid.

**Figure 11:**
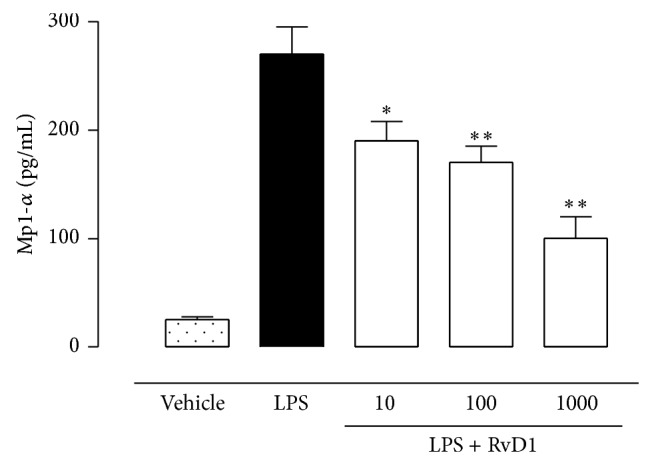
Intravitreal Resolvin D1 (RvD1) reduces the ocular content of MIP-1*α*. The ELISA assay shows decreased chemokine MIP1-*α* in the ocular tissues of LPS+RvD1 rats. Values are reported as mean ± SEM of *n* = 6 observation for each group. ^*^ *P* < 0.05 and ^**^ *P* < 0.01 versus LPS-treated rats.

The reduction of ocular inflammation in LPS+RvD1 treated rats was accompanied by an increase of M2 macrophage phenotype. Indeed, immunohistochemistry showed that RvD1 (10-100-1000 ng/kg) dose-dependently induced high expression of the macrophage M2 marker CD206 which was already significant (*P* < 0.01) at dose of 10 ng/kg (Figure 9). In contrast, there was a reduction of the macrophage M1 phenotype within the ocular tissue induced by RvD1, as evidenced by CD11c expression (Figure 10). Eye tissues of LPS+RvD1 also showed the lowest levels of the MIP-1*α* chemokine (Figure 11).

## 5. Discussion

Inflammation is terminated by endogenous anti-inflammatory and proresolving mediators aimed to restore cellular homeostasis [13]. Resolvins are classes from eicosapentaenoic and docosahexaenoic acids derived proteins denoted as E and D series, respectively, that play a pivotal role in the resolutive phase of inflammation. Resolvin D1 is the major component of these classes, it is produced physiologically during inflammatory process and it has scavenging effects on cytokines and chemokines [14–17], inhibiting the* de novo* production of cytokines and chemokines, the leukocytes trafficking/infiltration to inflamed tissue, and the production of PMN-derived free radicals [15, 16]. RvD1 has been involved in the resolution of several inflammatory pathologies [18], including uveitis [4]. However, in this latter pathology the mechanism through which RvD1 protects from ocular damage is not fully elucidated. Here we show that the administration of RvD1 into the vitreous of rats with LPS-induced endotoxic uveitis decreases the ocular damage through involvement of B and T lymphocytes, miRNAs, ubiquitin-proteasome system, and macrophages. To our knowledge, although the lymphocytes involvement in the RvD1 protection has been already described the miRNAs, ubiquitin-proteasome system and macrophages as new actors of the uveitic scenario is novelty. miRNAs are a small class of endogenous noncoding single-stranded RNA molecules (approximately 21–25 nucleotides) [19] that modulate gene expression at posttranscriptional level in animals and plants by targeting mRNAs for degradation or by inhibiting translation [20–22]. miRNAs as regulators of gene expression are implicated in several biological pathways, [23–27], in several autoimmune diseases and have anti-inflammatory or proinflammatory activities based on their specific target mRNAs [22, 26]. Using the real-time RT-PCR Array (qRT-pcr Array) [28], we show that there is an upregulation of 4 principal miRNAs into the eye of LPS+RvD1 rats with respect to the LPS alone 24 h after intravitreal administration of the protein. These were miR-21-5p that is predicted to regulate NFk-B activity; miR-200c-3p that is predicted to negatively regulate IL-13, LEPR, NTF3, PRKC*α*, RIPK2, and VEGFA indicating decreased of proinflammatory cytokines [29]; miR-203a-3p predicted to regulate IL-24 and PRKC*α*; miR-29b-3p predicted to negatively regulate HDAC4, IL-1RAP, Lif, PDGF*α*, PDGFc, VEGFA, and TNFRSF1. Overall, these miRNAs were relevant to the inflammatory response into the eye as our data show that in parallel with the changes in miRNAs RvD1 decreased TNF-*α* levels and NF-*κ*B expression into the eye. In addition, we report here that RvD1 treatment in LPS rats shifts the ocular resident macrophages from the M1 phenotype, most abundant in LPS-rats, to M2 phenotype, most abundant in LPS+RvD1-rats. Increasing the presence of M2 macrophages into the eye structure may favor the resolution of the ocular damage. Indeed M1 and M2 macrophages display different control of the inflammatory process, the M1 phenotype immediately after neutrophils invade the damaged tissue and has inflammatory function and phagocyte function, while the M2 phenotype has anti-inflammatory function through production of anti-inflammatory cytokines such as IL-4, IL-16, and IL-12 and activates the stem component [30]. These two phenotypes are differentially expressed following inflammatory stimulus (M1) such LPS and/or IFN-alpha [31] or anti-inflammatory stimulus (M2) such as IL-10, IL-13, IL-14, and glucocorticoids [32]. Moreover, it is well known that macrophages express ubiquitin-proteasome system (UPS) and their activities are regulated by this system [33, 34]. Interestingly, we show that the intravitreal administration of resolvin D1 in LPS-rats also causes reduction of the ubiquitin-proteasome system expression within the eye. This system is the major system for nonlysosomal intracellular protein degradation in eukaryotic cells and it is involved in a number of biological processes, including inflammation, proliferation, and apoptosis, that are responsible for progression of diseases to poor prognosis [35]. The ubiquitin-mediated proteolytic pathway involves cellular proteins in a multienzymatic process targeting proteins to degradation [36]. This ligation of ubiquitin by a series of ubiquitin-conjugating enzymes produces polyubiquitin chains, which serve as targeting signals for degradation of the protein by the proteasome. The multicatalytic proteasome consists of a central catalytic core, the 20S proteasome, and two regulatory 19S and 26S complexes [36]. Moreover, the ubiquitin-proteasome system is required for activation of nuclear factor kappa B (NF-*κ*B), a central transcription factor that regulates inflammatory genes, by degradation of its inhibitory kappa B (I*κ*B) proteins [37] and a series of downstream events leading to the development of the inflammatory responses within organs and tissues. No better knowledge of how RvD1 may reduce ubiquitin-proteasome expression is provided here; however, it is intriguing to speculate a proresolutive action of RvD1 in uveitis trough a less proteasome production from M1 macrophages shifted towards the M2 phenotype.

In conclusion, our study demonstrated that intravitreal administration of resolvin D1 in rats with endotoxic uveitis protects the eye from the damage caused by systemic LPS reducing the presence of B and T lymphocytes within the eye, changing the expression of some miRNAs, the polarization of the local macrophages, and decreasing the local levels of ubiquitin-proteasome.
